# Characterization and robust nature of newly isolated oleaginous marine yeast *Rhodosporidium* spp. from coastal water of Northern China

**DOI:** 10.1186/s13568-017-0329-x

**Published:** 2017-02-01

**Authors:** Qiuzhen Wang, Yan Cui, Biswarup Sen, Wenmeng Ma, Rose Lynn Zheng, Xianhua Liu, Guangyi Wang

**Affiliations:** 1 Center for Marine Environmental Ecology, School of Environmental Science and Engineering, Tianjin University, Tianjin, 300072 China; 2 School of Environment and Energy, Peking University Shenzhen Graduate School, Shenzhen, 518055 China

**Keywords:** Oleaginous yeast, Lipid production, Culture optimization, Fatty acid profile

## Abstract

A total of ten marine yeast strains isolated from Bohai Sea, Northern China were identified to be members of three genera *Rhodosporidium*, *Rhodotorula*, and *Cryptococcus*. Two representative strains *Rhodosporidium* TJUWZ4 and *Cryptococcus* TJUWZA11 with high lipid content based on Nile red staining method were further characterized. A wide range of culture conditions (C and N sources, pH, temperature, salinity and C/N ratio) were tested to characterize the biomass and lipid production (yield and productivity) of these strains. Results indicated that *Rhodosporidium* TJUWZ4 was capable of achieving lipid yield up to 44% and 0.09 g/l–h productivity on glucose and peptone medium at pH 4, 20 °C, 30% salinity, and C/N 80. Three fatty acids, namely oleic acid (18:1), palmitic acid (C16:0) and linoleic acid (18:2) were the major intracellular fatty acids, which accounted for 90% of total lipids. With promising features for intracellular lipid accumulation, *Rhodosporidium* TJUWZ4 is a robust strain with great potentials for application in biodiesel production from renewable feedstocks.

**Electronic supplementary material:**

The online version of this article (doi:10.1186/s13568-017-0329-x) contains supplementary material, which is available to authorized users.

## Introduction

Over last several decades, terrestrial yeasts have been widely studied for various applications in food, pharmaceutical, cosmetic and chemical industries (Kutty and Philip 2008). However, investigations on marine yeasts are comparatively few. Recent research revealed that marine yeasts own unique and promising features over their terrestrial counterparts, for example, higher osmosis tolerance, higher special chemical productivity and production of industrial enzymes (Zaky et al. 2014). Besides, oleaginous yeast have numerous advantages which make them promising sources of oil for biodiesel production (Ageitos et al. 2011). The wide features of oleaginous yeast make them a potent source of oil for sustainable development of bioenergy technology especially in the coastal regions around the globe.

Unlike other oil-producing microorganisms, such as microalgae, oleaginous yeast do not require long fermentation period and their resulting lipid profiles could be simply manipulated by varying the fermentation conditions (Dias et al. 2015; Sitepu et al. 2014). In addition, oleaginous yeast can grow on various substrates, even inexpensive materials such as organic wastes, thus making the lipid production more efficient from low-cost raw materials (Deeba et al. 2016; Ghanavati et al. 2015; Polburee et al. 2015; Slininger et al. 2016; Xiong et al. 2015; Zhang et al. 2016). The lipids accumulated by oleaginous yeast are mainly composed of long-chain fatty acids, including oleic acid (C18:1), palmitic acid (C16:0), linoleic acid (C18:2) and stearic acid (C18:0), which are similar to the composition of plant oils and can be converted into biodiesel by enzymatic or inorganic catalysis (Ghanavati et al. 2014; Spier et al. 2015; Tanimura et al. 2014a; Wang and Ren 2014). Thus, much attention have been paid to various oleaginous yeasts for biodiesel production (Areesirisuk et al. 2015; Deeba et al. 2016; Poli et al. 2014; Seo et al. 2014; Sitepu et al. 2014; Spier et al. 2015; Tanimura et al. 2014a, b; Yang et al. 2014).

Both yeast strains and culture conditions have great impacts on lipid accumulation and fatty acid profiles (Rakicka et al. 2015; Sitepu et al. 2013). Total lipids and fatty acid profiles vary significantly between species, to some extent among strains of the same species, also for the same strain grown under different culture conditions such as carbon source, nitrogen source, temperature, pH, salt concentration and C/N ratio (Béligon et al. 2015; Braunwald et al. 2013; Cescut et al. 2014). Sugar-based media such as glucose, xylose, fructose, lactose, starch, and lignocellulosic hydrolyzates have been studied for lipid production, but glucose was found to be more easily metabolized compared to other substrates (Papanikolaou and Aggelis 2011). Glucose and glucose containing substrates are thus the most common carbon sources used for growth and lipid production by oleaginous yeast.

For lipid accumulation, the crucial factor is the change of intracellular concentration of certain metabolites due to nitrogen limitation in culture medium. The influence of carbon-to-nitrogen (C/N) ratio on lipid accumulation by *Rhodotorula glutinis* was investigated, and showed increased lipid production at higher C/N ratios (Braunwald et al. 2013). Another study with *Lipomyces starkeyi* cultivated in a medium based on sewage sludge supplemented with glucose showed 68% lipid accumulation at C/N ratio of 150, and a lipid content of 40% at C/N ratio of 60 (Angerbauer et al. 2008). These reports signify nitrogen limitation as an important criterion for higher lipid accumulation. Besides C/N ratio, pH also strongly influences lipid accumulation. In *Rhodotorula glutinis*, notable difference in lipid yield (%) was observed at pH 3 (12%), pH 5 (48%) and pH 6 (44%) (Johnson et al. 1992).

From above studies it is evident that lipid yields are influenced to a great extent by the media composition (carbon source, nitrogen source, minerals etc.), pH and temperature. In this study, marine yeast strains were isolated from the coastal water of North China, and the representative strains characterized for their ability to produce lipids under different fermentation conditions including carbon source, nitrogen source, temperature, pH, salt concentration and C/N ratio.

## Materials and methods

### Sample collection and strain isolation

Seawater samples were collected from coastal waters of Bohai Bay in North China and were then plated on M4 solid medium (glucose·H_2_O 10 g/l, peptone 1.5 g/l, yeast extract 0.1 g/l, and agar 20 g/l) at room temperature for 3–5 days with regular observation for yeast growth. The obtained cell colonies were then inoculated in M4 solid medium containing 0.05% ampicillin for axenic cultures. Pure isolates were maintained at 28 °C on YEPD medium (glucose·H_2_O 20 g/l, peptone 10 g/l, yeast extract 10 g/l, and agar 15 g/l) (Shen et al. 2013) and sub-cultured every 20–30 days.

### Identification of yeast strains

Yeast isolates were identified by sequence analysis of 18S rRNA gene. Total genomic DNA of yeast cells was extracted using DNA extraction kit (Generay, China), following the manufacturer’s instruction. The 18S rRNA gene fragment was amplified by polymerase chain reaction (PCR) in the S1000™ Thermal cycler (Bio-Rad, USA) with the fungi universal primers, EF4 (5′-GGAAGGG (G/A) TGTATTTATTAG-3′) and EF3 (5′-TCCT (A/C) TAAATGACCAAGTTTG-3′) (Smit et al. 1999). The PCR reaction was carried out under the initial denaturation at 94 °C for 5 min, followed by 30 cycles of 30 s at 94 °C, 30 s at 51 °C and 90 s at 72 °C, and a final extension at 72 °C for 10 min. The PCR products were purified using Gel DNA extraction kit (Generay, China). The resulting PCR products were sequenced at Beijing Genomics Institute (China) and the final sequences were edited using BioEdit and then compared with the National Center for Biotechnology Information database. Phylogenetic analyses were carried out using distance setting (Maximum likelihood) in MEGA 6 software with 1000 bootstrap replicates (Tamura et al. 2013). The 18S rRNA gene sequences obtained for this study were submitted to GenBank under the access number of KU317620-317626 and KT281890-281892.

### Strain screening with Nile red method

The marine yeast strains were screened for lipid production using the Nile red staining method (Li et al. 2014). Yeast cells were pre-cultured in 100 ml flasks containing 50 ml basic broth medium-A on an incubator shaker at 200 rpm and 30 °C for 5 days. One liter of the medium-A contained: glucose 120 g, CaCl_2_ 0.1 g, NH_4_Cl 0.5 g, yeast extract 1.5 g, KH_2_PO_4_ 7.0 g, Na_2_HPO_4_·2H_2_O 2.5 g, MgSO4·7H_2_O 1.5 g, FeCl_3_·6H_2_O 0.08 g, ZnSO_4_·7H_2_O 10.0 mg, MnSO_4_·H_2_O 0.07 mg, CuSO_4_ 0.1 mg, Co(NO_3_)_2_ 0.063 mg (Sitepu et al. 2013). At 24 h intervals, an aliquot of the culture was sampled and centrifuged at 4000 rpm for 10 min. The resulting cell pellet was washed twice with sterile seawater and then stained with 0.01% (w/v) Nile Red in acetone. The intracellular lipids were examined by using an Epifluorescence Microscope (Nikon DS-Ri1) at the wavelength of 540 nm. Strains with high lipid accumulation exhibit high intensity of fluorescence. Based on this characteristic, lipid-producing strains were selected for further investigation of lipid production and fermentation optimization. The promising strain *Rhodosporidium* sp. TJUWZ4 has been deposited in China General Microbiological Culture Collection Center (CGMCC # 2.5689).

### Optimization of culture conditions

Six different parameters were investigated for their individual effect on cellular growth and lipid production of the representative strains screened by Nile red method. These variables included carbon source (glucose, sucrose, fructose, lactose and starch), nitrogen source (yeast extract, peptone, NH_4_Cl, (NH_4_)_2_SO_4_ and mixture of both of them), C/N ratio (20C, 20 N, 80, 120C, 120 N), initial pH of the medium (3.0, 4.0, 5.0, 6.0, and 7.0), temperature (15, 20, 25, 30 and 35 °C) and salinity strength (30, 50, 80 and 100%, v/v). The six sets of single-factor experiments were designed by varying the broth medium-A and each subsequent experiment was based on previous optimal results obtained. The experimental design and conditions are shown in Additional file 1: Table S1. The first set experiment was to determine the optimal carbon source (sugar). Individual sugar was added to a concentration of 120 g/l for each treatment to determine the best sugar as the sole carbon source. Then optimal nitrogen source and concentrations were evaluated with the best carbon source. The best carbon and nitrogen source obtained were applied to investigate the optimum pH value. The pH was adjusted by using sulfuric acid and sodium hydroxide solutions. Accordingly, the temperature, salinity strength and C/N ratio were tested at the optimal pH value. Salinity strength was defined by seawater concentration adjusted by artificial sea salt in this study.

For all sets of experiment, the yeast isolates were cultured in 100 ml Erlenmeyer flasks containing 50 ml broth medium-A for 60 h at room temperature with shaking at 200 rpm. All experiments were conducted in triplicates, and the results were expressed as means of the replicates along with standard deviation (±SD).

### Analytical methods

At the end of the experiment, yeast cells were harvested by centrifugation at 4000 rpm for 10 min from 15 ml culture broth, washed twice with sterile distilled water and lyophilized for 48 h using a freeze-drying system (Christ, USA). The biomass concentration was expressed as dry cell weight per liter. Lipid yield was calculated as g lipid per g biomass, and productivity (g/l–h) as lipid production (g/l) over time of incubation (60 h).

Total lipids of yeast strains were extracted using the direct transesterification method described elsewhere (Lepage and Roy 1984). Freeze-dried cells (50–100 mg) and 100 μl of 1.0 mg/ml internal standard (TAG 17:0 (glyceryl trihepta-decanoate), catalogue no. T2151, Sigma-Aldrich Co. LLC., USA) solution were dissolved directly in two ml of 4% sulfuric acid in methanol, vortexed for 30 s, and were then methyl-esterified at 80 °C for 1 h. To extract the FAMEs, one ml each of water and hexane were added to the mixtures at room temperature. After vortexing and centrifuging at 4000 rpm for 5 min, the fatty acids converted into fatty acid methyl esters (FAMEs) in the hexane layer was analyzed using GC–MS (Agilent 7890A-5975C, USA) equipped with a HP-5 ms capillary column (30 m × 250 μm × 0.25 μm). Helium was used as the carrier gas with a flow of 0.8 ml/min. A volume of 1.0 μl sample was injected into GC–MS, using a 10.0 μl syringe in splitless injection mode. The injection port temperature was set to 240 °C. The initial column temperature was maintained at 150 °C for 2 min followed by programming at 4 °C/min to 230 °C and held for 5 min.

## Results

### Oleaginous yeast strain isolation and molecular identification

**Fig. 1:**
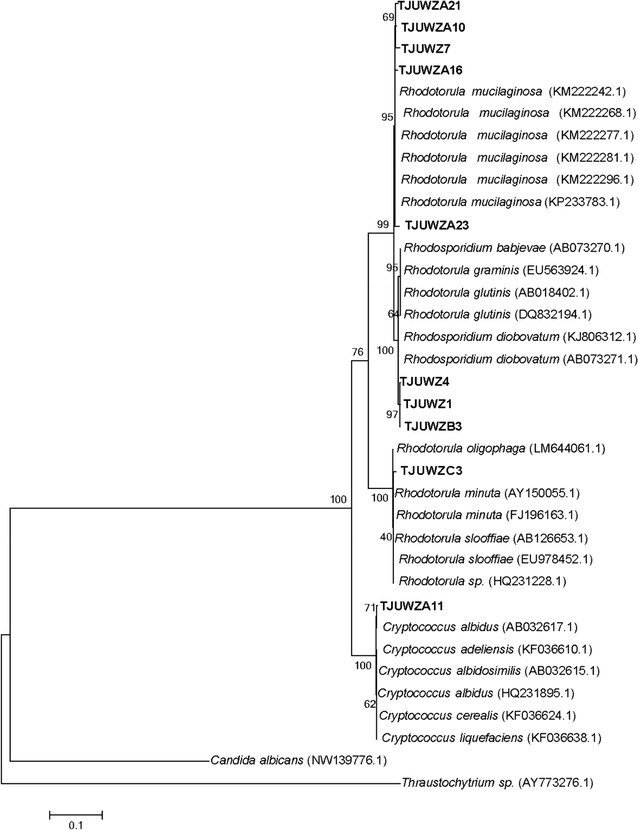
Phylogenetic tree of marine yeast strains isolated from Bohai Bay of North China. Maximum likelihood tree was built using Mega 6 based on 18S rRNA genes from 10 marine yeast strains with sequences from *Candida albicans* and *Thraustochytrium* sp. as outgroups. Numbers above or below branches indicate bootstrap values (>50%) from 1000 replications

Ten marine yeast strains were isolated from coastal water of northern China. Colonies of all strains were observed to be red except TJUWZA11 with white appearance. Microscopy showed spherical to ovoid cells with the size ranging from 2 to 8 μm. For molecular identification and phylogenetic analyses, their 18S rRNA gene fragments were amplified from the total genomic DNA using the universal primers and subjected to sequencing analysis. Maximum likelihood tree was built based on the evolutionary distance of ten yeast strains with *Candida albicans* and *Thraustochytrium* sp. as outgroups (Fig. 1). Phylogenetic analyses indicated that these 10 yeast strains belonged to three genera of yeasts i.e., *Rhodotorula*, *Rhodosporidium* and *Cryptococcus*.

### Screening of oleaginous yeast strains with Nile red method

**Fig. 2:**
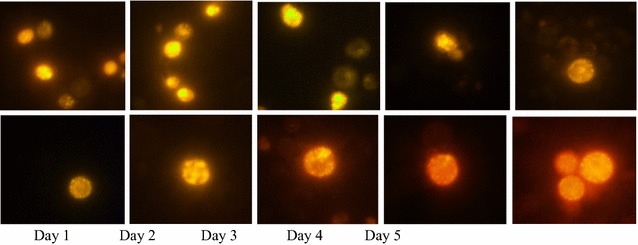
Epifluorescence microscopy of the Nile Red stained isolate *Rhodosporidium* TJUWZ4 (*top panel*) and *Cryptococcus* TJUWZA11 (*bottom panel*) at different stages of growth

Nile red is a lipid-soluble dye and has been widely employed to determine the cellular lipid content in qualitative analysis (Kimura et al. 2004). Among all the strains tested, *Rhodosporidium* TJUWZ4 and *Cryptococcus* TJUWZA11 showed a significant amount of lipid accumulation in shake flasks by exhibiting positive Nile red staining. The intensity of fluorescence for stained intracellular lipids increased with the ages of cultivation, reaching maximum on the 2nd day of cultures with golden yellow fluorescence as shown in Fig. 2. Since both strains exhibited highest fluorescence on the second day of cultivation, the subsequent optimization of cultivation conditions for lipid production and analysis were done at 60 h. The specific growth rates determined from the growth curves of *Rhodosporidium* TJUWZ4 and *Cryptococcus* TJUWZA11 were 0.026 and 0.035 h^−1^, repectively.

### Effect of carbon and nitrogen sources on biomass production and lipid yield

**Fig. 3:**
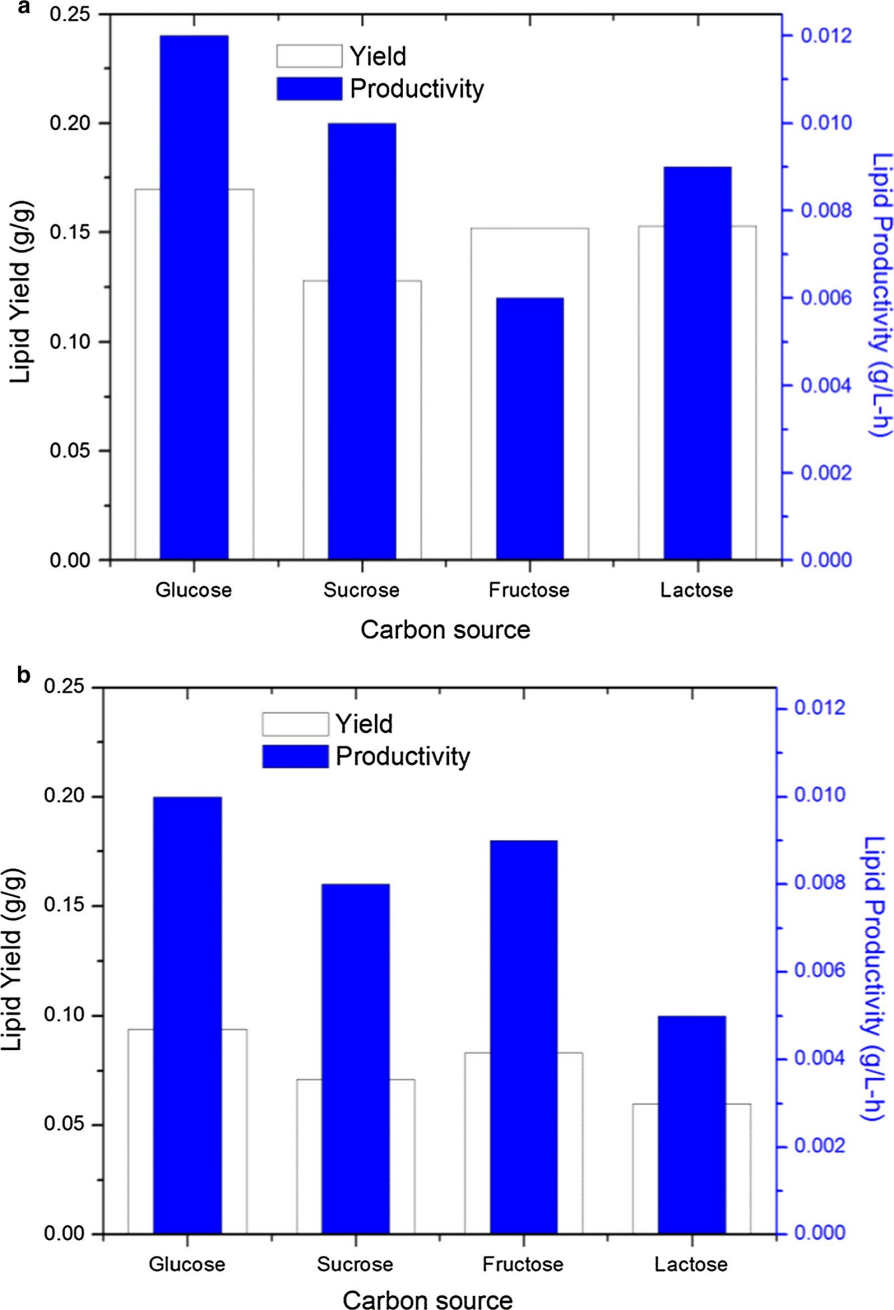
Effect of various carbon sources on lipid yield of **a** *Rhodosporidium* TJUWZ4 and **b** *Cryptococcus* TJUWZA11

Two monosaccharides (glucose and fructose), two disaccharides (sucrose and lactose) and one polysaccharide (starch) were used to investigate the influence of carbon source on cellular growth and lipid production in flasks. As shown in Table 1, for a given initial sugar concentration of 120 g/l, lipid accumulation was found in almost all carbon sources except for the very low lipid content (0.039 g/l) in *Rhodosporidium* TJUWZ4 grown on starch. Glucose was shown to be the best substrate with maximum lipid concentration of 0.73 and 0.62 g/l for *Rhodosporidium* TJUWZ4 and *Cryptococcus* TJUWZA11, respectively. The biomass was slightly higher on sucrose as compared to other carbon sources for both strains. The maximum lipid yield (0.17 g/g and 0.09 g/g for TJUWZ4 and TJUWZA11, respectively) and productivity (0.012 g/l–h and 0.01 g/l–h for TJUWZ4 and TJUWZA11, respectively) were found on glucose (Fig. 3a, b).

**Table 1:** Effect of carbon and nitrogen sources on biomass and lipid production in newly isolated marine oleaginous yeast strains

Treatment	*Rhodosporidium* TJUWZ4		*Cryptococcus* TJUWZA11	
	Biomass (g/L)	Lipid (g/L)	Biomass (g/L)	Lipid (g/L)
Carbon source				
Glucose	4.29 ± 0.08	0.73 ± 0.04	6.57 ± 0.15	0.62 ± 0.02
Sucrose	4.70 ± 0.11	0.60 ± 0.11	6.94 ± 0.13	0.49 ± 0.07
Fructose	2.30 ± 0.14	0.35 ± 0.04	6.30 ± 0.16	0.52 ± 0.03
Lactose	3.66 ± 0.27	0.56 ± 0.05	5.04 ± 0.04	0.30 ± 0.01
Starch	ND	0.039 ± 0.00	ND	0.41 ± 0.02
Nitrogen source				
Peptone	5.39 ± 0.49	1.37 ± 0.07	6.42 ± 0.08	0.75 ± 0.02
Yeast extract	7.65 ± 0.07	1.19 ± 0.27	6.73 ± 0.13	0.66 ± 0.02
NH_4_Cl	6.1 ± 0.32	0.88 ± 0.08	4.25 ± 0.15	0.45 ± 0.01
(NH4)_2_SO_4_	4.71 ± 0.04	0.75 ± 0.06	4.14 ± 0.11	0.38 ± 0.01
(NH4)_2_SO_4_ + yeast extract	6.92 ± 0.34	1.21 ± 0.14	7.45 ± 0.09	0.74 ± 0.08
(NH4)_2_SO_4_ + peptone	5.25 ± 0.18	0.90 ± 0.02	6.00 ± 0.19	0.60 ± 0.1
NH_4_Cl + yeast extract	5.00 ± 0.34	0.64 ± 0.09	5.62 ± 0.02	0.45 ± 0.08
NH_4_Cl +peptone	5.42 ± 0.57	0.90 ± 0.11	5.30 ± 0.35	0.38 ± 0.05
Yeast extract + peptone	7.35 ± 0.42	1.52 ± 0.08	7.36 ± 0.06	0.69 ± 0.07

*ND* not determined

The experimental results indicated that *Cryptococcus* TJUWZA11 could utilize all carbon sources while *Rhodosporidium* TJUWZ4 could not accumulate lipids in the starchy medium. Previous report on *Cryptococcus terricola* also showed accumulation of high lipid content up to 61.96% on medium with 5% starch after a 10-day growth period (Tanimura et al. 2014a). To grow on starchy medium, *Cryptococcus terricola* could degrade starch to oligosaccharides by using their own extracellular amylases. It was therefore suggested that starch was not assimilated directly by most yeast and required extracellular enzymes. In the present study the biomass content of the two strains grown on starch could not be determined owing to the infeasibility of removing the residual starch in the culture after fermentation.

**Fig. 4:**
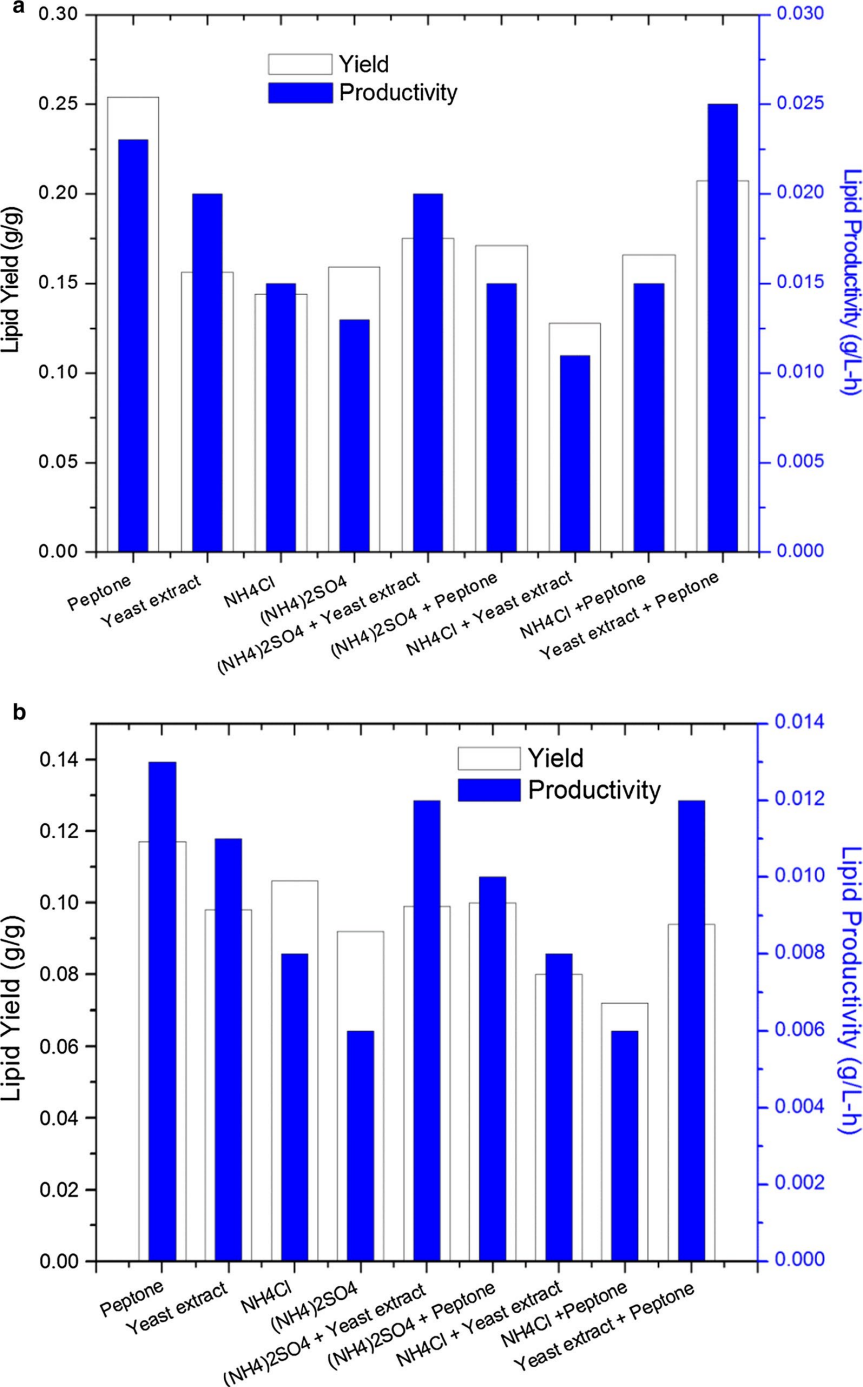
Effect of various nitrogen sources on lipid yield of **a** *Rhodosporidium* TJUWZ4 and **b** *Cryptococcus* TJUWZA11

The influence of four different nitrogen sources, namely yeast extract, peptone, NH_4_Cl and (NH_4_)_2_SO_4_, on biomass and lipid production is shown in Table 1. Results indicated that all nitrogen sources were able to support growth but the biomass and lipid concentration showed great differences. When organic nitrogen (peptone or yeast extract) was used as the sole nitrogen source for yeast growth, the lipid concentration was 1.37 and 0.75 g/l in peptone, or 1.19 and 0.66 g/l in yeast extract, for stain TJUWZ4 and TJUWZA11 respectively, which were much higher than the values obtained in inorganic sources. The biomass concentration was highest (7.65 g/l) on yeast extract for TJUWZ4, but for TJUWZA11 a combination of (NH_4_)_2_SO_4_ and yeast extract was required to achieve the maximum value of 7.45 g/l. Among all nitrogen sources used, peptone alone showed maximum lipid yield (0.254 and 0.117 g/g) and productivity (0.023 and 0.013 g/l–h) for TJUWZ4 and TJUWZA11 respectively (Figs. 3a, 4b). In cases of organic source mixed with inorganic nitrogen source (NH_4_Cl + yeast extract, NH_4_Cl + peptone, (NH_4_)_2_SO_4_ + yeast extract, (NH_4_)_2_SO_4_ + peptone), the lipid yield and productivity were lower than in sole organic source, but higher than in sole inorganic source. A previous study suggested that the vitamins and amino acids contained in organic sources could facilitate the growth of yeast cells thus increasing the lipid yield (Kitcha and Cheirsilp 2013). These results indicate that organic nitrogen sources are more beneficial for lipid production by marine yeast when compared with inorganic sources.

### Effect of pH, temperature and salinity of medium on lipid yield

**Fig. 5:**
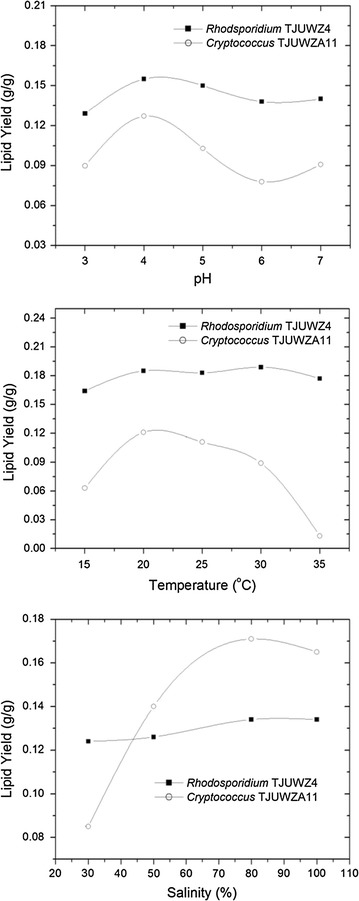
Lipid yields pattern of the newly isolated oleaginous yeast strains in response to variation in pH, temperature and salinity

While the effects of carbon source, nitrogen source and their ratios on lipid production have been extensively examined, other factors such as pH, temperature and salinity have not been well studied to optimize the process parameters. In our study, both strains were capable of accumulating lipid in the broths with the initial pH range of 3–7 (Table 2), exhibiting acid tolerance property. The lipid concentration ranged from 0.84 to 1.02 g/l for TJUWZ4, and from 0.42 to 0.66 g/l for TJUWZA11 within the experimental pH range. At pH 4 the lipid yield and productivity reached maximum level (Fig. 5). The highest lipid yield for TJUWZ4 and TJUWZA11 strains were 0.155 and 0.127 g/g with productivity of 0.017 and 0.011 g/l–h, respectively (Fig. 5). The ability of these two strains to accumulate high lipids at low pH makes them ideal candidates for continuous and semi-continuous pilot scale operations, as low pH discourages growth of contaminating bacteria.

**Table 2:** Effect of pH, temperature and salinity on biomass and lipid production of newly isolated oleaginous yeast strains

Treatment	*Rhodosporidium* TJUWZ4		*Cryptococcus* TJUWZA11	
	Biomass (g/l)	Lipid (g/l)	Biomass (g/l)	Lipid (g/l)
pH				
3	6.49 ± 0.31	0.84 ± 0.10	4.66 ± 0.18	0.42 ± 0.02
4	6.56 ± 0.21	1.02 ± 0.11	5.18 ± 0.26	0.66 ± 0.03
5	6.00 ± 0.07	0.90 ± 0.12	6.12 ± 0.07	0.63 ± 0.06
6	6.50 ± 0.25	0.90 ± 0.12	7.58 ± 0.29	0.59 ± 0.05
7	6.73 ± 0.05	0.94 ± 0.02	6.02 ± 0.12	0.55 ± 0.04
Temperature				
15	6.42 ± 0.28	1.05 ± 0.09	4.60 ± 0.23	0.29 ± 0.02
20	7.96 ± 0.58	1.47 ± 0.05	6.42 ± 0.21	0.78 ± 0.03
25	8.03 ± 0.64	1.47 ± 0.04	6.82 ± 0.04	0.76 ± 0.06
30	5.88 ± 1.18	1.11 ± 0.00	7.52 ± 0.56	0.67 ± 0.07
35	3.55 ± 0.23	0.63 ± 0.02	0.76 ± 0.22	0.01 ± 0.00
Salinity				
0	7.14 ± 0.32	0.83 ± 0.01	7.45 ± 0.18	1.17 ± 0.01
30	7.10 ± 0.26	0.88 ± 0.05	7.78 ± 0.45	0.66 ± 0.04
50	6.10 ± 0.34	0.77 ± 0.09	6.84 ± 0.22	0.96 ± 0.14
80	5.91 ± 0.21	0.79 ± 0.03	7.00 ± 0.26	1.20 ± 0.05
100	5.98 ± 0.49	0.80 ± 0.07	6.35 ± 0.06	1.05 ± 0.10

The effect of temperature on biomass and lipid production of the two newly isolated oleaginous yeast strains is shown in Table 2. An increase in temperature from 30 to 35 °C led to a drop in biomass and lipid production for both strains. At 35 °C, there was a sharp decrease in the growth of *Cryptococcus* TJUWZA11 unlike *Rhodosporidium* TJUWZ4. Similarly there was a decline in biomass and lipid production when strains were grown at temperature below 20 °C. However, the drop in biomass and lipid production in *Cryptococcus* TJUWZA11 was of greater magnitude than *Rhodosporidium* TJUWZ4. The lipid productivity were maximum at temperature range of 20–25 °C with 0.025 and 0.013 g/l–h for *Rhodosporidium* TJUWZ4 and *Cryptococcus* TJUWZA11, respectively (Fig. 5). The lipid yield was highest at 30 °C for *Rhodosporidium* TJUWZ4 (0.19 g/g) and 20 °C for *Cryptococcus* TJUWZA11 (0.12 g/g) (Fig. 5). *Rhodosporidium* TJUWZ4 exhibited wide range of temperature (20–30 °C) tolerance with high lipid yield (0.185–0.189 g/g) whereas *Cryptococcus* TJUWZA11 was found to be temperature sensitive and gave highest lipid yield (0.121 g/g) at 20 °C.

As shown in Table 2, both strains were able to grow and produce lipids at all levels of tested salinity (ranging from 30 to 100% of natural seawater). For *Cryptococcus* TJUWZA11, the optimal seawater concentration was 80%, under which the obtained lipid concentration and yield were 1.2 g/l and 0.17 g/g respectively. It was also found that the salinity strength had little effect on the biomass and lipid production (Table 2) and also on the lipid yield (Fig. 5) of *Rhodosporidium* TJUWZ4.

### Effect of carbon-to-nitrogen ratio on improving lipid yield

**Fig. 6:**
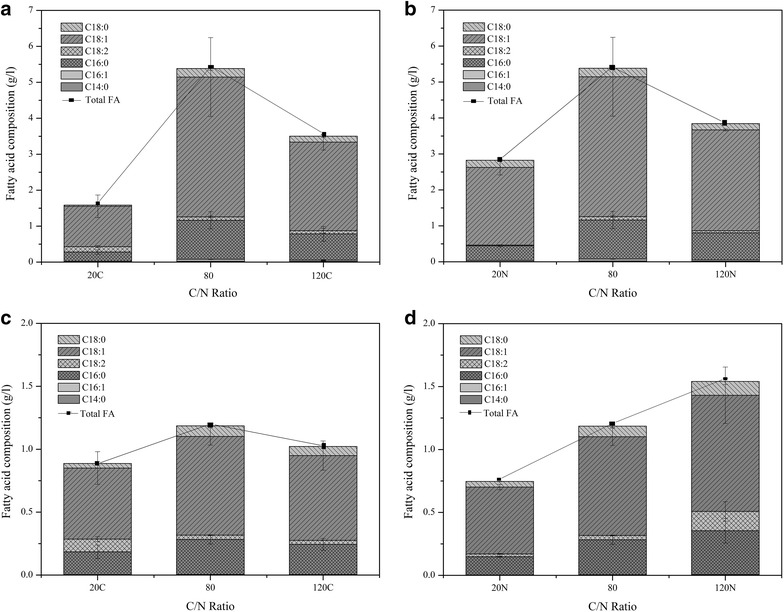
Lipid production and composition at different C/N ratios for *Rhodosporidium* TJUWZ4 (**a**, **b**) and *Cryptococcus* TJUWZA11 (**c**, **d**)

It is well known that in a medium with high C/N ratio oleaginous yeast exhibit high yield since after the exhaustion of nitrogen present in the medium the excess carbon is converted to lipid droplets and stored within the yeast cell. In this study, the best carbon and nitrogen sources for high lipid yield and productivity were glucose and peptone respectively for both strains. The C/N ratios used in the experiments were obtained by varying the concentration of added carbon source (glucose) and nitrogen source (peptone and yeast extract) in the medium as listed in Table 3. For the calculation of C/N ratios of 20 C and 120 C, the amount of nitrogen source was kept constant and the carbon source was varied and vice versa for C/N ratios of 20 N and 120 N. At different levels of initial glucose (Fig. 6a, c), yeast cells accumulated more lipid when the C/N ratio was raised from 20 C to 80. However, a further increase to C/N 120 C did not lead to higher lipid yields for both strains. On the other hand when C/N ratio was increased through nitrogen limitation (Fig. 6b, d), the isolates behaved differently. In *Rhodosporidium* TJUWZ4 the lipid production increased up to 5.4 g/l at C/N of 80 and with further increase in C/N to 120 the lipid yield dropped to 3.86 g/l. However, *Cryptococcus* TJUWZA11 showed increase in lipid production (1.54 g/l) with higher nitrogen limitation up to C/N 120 N. The maximum lipid yield and productivity were achieved at C/N 80 for *Rhodosporidium* TJUWZ4 whereas for *Cryptococcus* TJUWZA11 it was 120 N (Table 3). Among the two strains the highest lipid yield achieved was 0.44 g/g (44%) with productivity of 0.09 g/l–h by *Rhodosporidium* TJUWZ4.

**Table 3:** C/N ratios and their effects on biomass, lipid production, yield and productivity of newly isolated oleaginous yeast strains

Treatment	Glucose (g/l)	Peptone (g/l)	Yeast extract (g/l)	Biomass (g/l)	Lipid (g/l)	Yield (g/g)	Productivity (g/l–h)
*Rhodosporidium* TJUWZ4							
20C^a^	12.05	0.5	1.5	8.22 ± 0.64	1.60 ± 0.41	0.19	0.027
20N^b^	48	2	6	11.83 ± 0.22	2.86 ± 0.22	0.24	0.048
80	48	0.5	1.5	12.4 ± 0.78	5.40 ± 1.42	0.44	0.090
120C^a^	72.3	0.50	1.5	11.54 ± 0.54	3.52 ± 0.56	0.31	0.059
120N^b^	48	0.33	0.99	13.32 ± 1.19	3.86 ± 0.05	0.29	0.064
*Cryptococcus* TJUWZA11							
20C^a^	14	2	0	8.92 ± 0.73	0.91 ± 0.25	0.10	0.015
20N^b^	56	8	0	8.2 ± 0.43	0.75 ± 0.02	0.09	0.013
80	56	2	0	9.64 ± 0.41	1.19 ± 0.11	0.12	0.020
120C^a^	84	2	0	9.38 ± 0.81	1.02 ± 0.18	0.11	0.017
120N^b^	56	1.33	0	10.16 ± 0.78	1.54 ± 0.43	0.15	0.026

C/N calculation formula: $${{{\text{C}}/{\text{N}} = \left[ {\text{Glu}} \right] \times 0. 4 { }\left( {{\text{g}} - {\text{C}}/{\text{g}} - {\text{Glu}}} \right)} \mathord{\left/ {\vphantom {{{\text{C}}/{\text{N}} = \left[ {\text{Glu}} \right] \times 0. 4 { }\left( {{\text{g}} - {\text{C}}/{\text{g}} - {\text{Glu}}} \right)} {\left\{ {\left( {\left[ {\text{Pep}} \right]{\text{ x }}0. 1 4 { }\left( {{\text{g}} - {\text{N}}/{\text{g}} - {\text{Pep}}} \right)} \right) + \left( {\left[ {\text{YE}} \right]{\text{ x }}0. 1 1 4 { }\left( {{\text{g wt}}.{\text{ N in YE}}} \right)} \right)} \right\}}}} \right. \kern-0pt} {\left\{ {\left( {\left[ {\text{Pep}} \right]{\text{ x }}0. 1 4 { }\left( {{\text{g}} - {\text{N}}/{\text{g}} - {\text{Pep}}} \right)} \right) + \left( {\left[ {\text{YE}} \right]{\text{ x }}0. 1 1 4 { }\left( {{\text{g wt}}.{\text{ N in YE}}} \right)} \right)} \right\}}}$$

^a^C means that altering C/N ratio by changing carbon source contents at a constant concentration of nitrogen source

^b^N means that altering C/N ratio by changing nitrogen source contents at a constant concentration of carbon source

The fatty acid compositions of the lipid accumulated by both strains cultivated with different C/N ratios were similar with only minor variations in concentration (Table 4; Fig. 6). The main fatty acids were oleic acid (C18:1), which ranged from 59.9 to 76.5% of the total fatty acids, followed by palmitic acid (C16:0), stearic acid (18:0) and linoleic acid (18:2). Myristic acid (14:0) and palmitoleic acid (16:1) were only detected in trace amounts. Fatty acids with 16 and 18 carbon atoms comprised over 90% of the total fatty acids. The obtained fatty acid profiles were quite similar to previously published reports (Meesters et al. 1996) and also to those of plant oils, for instance, sunflower and canola oils (Ageitos et al. 2011). Sixty-nine strains representing 17 genera and 50 species were surveyed and it was found that the dominant fatty acids of the tested yeast strains were oleic (18:1), palmitic (16:0), stearic (18:0), and linoleic (18:2) acids (Sitepu et al. 2013). Minor fatty acids were lignoceric acid (24:0), palmitoleic acid (16:1), behenic acid (22:0), myristic acid (14:0), linolenic acid (18:3) and arachidic acid (22:0). In another work, twelve different yeast strains were evaluated for their lipid content and fatty acid profiles, and the data demonstrated that the predominant fatty acids are long chain fatty acids with 16–18 carbon atoms, including palmitic acid, oleic acid, linoleic acid and linolenic acid (Spier et al. 2015).

**Table 4:** Fatty acid composition produced by cultures of two newly isolated oleaginous yeast strains

Fatty acid	*Rhodosporidium* TJUWZ4 (%)	*Cryptococcus* TJUWZA11 (%)
Myristic acid C14:0	0.7	0.4
Palmitic acid C16:0	24.3	30.4
Palmitoleic acid C16:1	1.2	0.7
Stearic acid C18:0	3.1	3.7
Oleic acid C18:1	59.8	41.8
Linoleic acid C18:2	10.9	23.0
Total	100	100

## Discussion

Oleaginous yeast are known to accumulate lipids up to 20% of their biomass (Ageitos et al. 2011), thus both strains isolated in our study are oleaginous by this definition. Only 3–10% of the 1600 known yeast species are oleaginous, and the identified genera include *Yarrowia*, *Candida*, *Rhodotorula*, *Rhodosporidium*, *Cryptococcus*, *Trichosporon* and *Lipomyces* (Sitepu et al. 2014). Based on the 18S rRNA analysis, the isolated strains TJUWZ4 and TJUWZA11 belonged to *Rhodosporidium* and *Cryptococcus* genera, respectively, and their ability for lipid accumulation has been demonstrated to be consistent with previous published results (Meesters et al. 1996; Sitepu et al. 2013, 2014).

Oleaginous yeast capable of producing high lipid titers and yield is crucial to the bioprocess for conversion of lignocellulosic waste to lipids, which can further be converted to biodiesel (Slininger et al. 2016). A robust strain exhibiting a wide range of adaptability and tolerance to the growth conditions would be an ideal candidate for development of such bioprocess. In the present study one of the isolates *Rhodosporidium* TJUWZ4 was found to exhibit such desired properties. It exhibited acid, temperature and salinity tolerance and gave highest lipid yield up to 44% (0.4 g/g) of cellular dry weight at C/N 80. It is noteworthy that only 5% of reported oleaginous yeasts can accumulate more than 25% of lipids (Ageitos et al. 2011), and our results of *Rhodosporidium* TJUWZ4 has shown much higher than 25% placing it among the highest lipid accumulating oleaginous yeast.

While lipid content and yield can vary greatly among species, overall fatty acid profiles have been shown to be quite consistent under all conditions. As shown in Table 4, the major fatty acids of two tested strains were oleic acid (ca. 60% in TJUWZ4 and ca. 42% in TJUWZA11), palmitic acid (24.3% in TJUWZ4 and 30.4% in TJUWZA11), and linoleic acid (ca. 11% in TJUWZ4 and 23% in TJUWZA11). Minor fatty acids were stearic acid (18:0), myristic acid (14:0) and palmitoleic acid (16:1). These are consistent with the range of lipids generally found in other oleaginous yeast species (Dias et al. 2015; Sitepu et al. 2013).

Fatty acid composition has significant impacts on performance of biodiesel. Major properties of any biodiesel that are directly influenced by the FAME composition include: cetane number (CN), melting point, oxidative stability, kinematic viscosity and heat of combustion, which should be modified to comply with official standards ASTM D6751 and EN 14214 (Kaneko et al. 1976). Based on the fatty acid profiles observed in our study, it can be suggested that lipids from either yeast could be ideal candidates for biodiesel production purposes. However, owing to the robust nature of *Rhodsporidium* TJUWZ4, to changes in culture conditions, it could be a potential oleaginous yeast strain for biodiesel production in pilot scale oil production under batch as well as semi-continuous mode.

Lipid accumulation normally takes places when nitrogen source is depleted from the medium while carbon source is still present in high amounts (Gao et al. 2014; Granger et al. 1993). Under this condition, the excess carbon source is channeled into lipid bodies to form triglycerides (TAGs). From this, it can be deduced that a high C/N ratio of the media would be favorable for lipid accumulation. Accordingly, the lipid yield was increased through the nitrogen limitation. Our results also implied that at a certain level of nitrogen source, an increase of carbon loadings (glucose in the case) from 48 g/l (C/N 80) to 72.3 g/l (C/N 120C) was not beneficial for the lipid production. There existed an optimum initial C/N ratio, and when the value of C/N ratio was higher or lower than the optimal one, lipid accumulation decreased. Therefore, utilization of carbon sources should be controlled in the batch fermentation since carbon accounts for a large amount of the total production cost, and any potential saving in carbon utilization will help to reduce the processing cost and realize the economic industrial application.

In conclusion, ten marine yeast strains were isolated from Bohai Sea of northern China. Sequence analyses indicated that they belonged to three genera: *Rhodosporidium*, *Rhodotorula*, and *Cryptococcus*. Lipid production analyses identified two high lipid-producing strains, namely *Rhodosporidium* TJUWZ4 and *Cryptococcus* TJUWZA11. Further characterization of these two strains for their ability to accumulate lipid under various culture conditions revealed that *Rhodosporidium* TJUWZ4 (44% yield on glucose and peptone with C/N 80) is among the 5% reported oleaginous yeast able to accumulate above 25% lipid. Moreover, *Rhodosporidium* TJUWZ4 showed tolerance to a wide range of pH, temperature and salinity. The predominant fatty acid profiles were oleic acid (18:1), palmitic acid (C16:0) and linoleic acid (18:2) accounting to 90% of total fatty acids, highly desirable for better biodiesel properties. Thus, *Rhodosporidium* TJUWZ4 has great potentials for application in microbial based biodiesel production, and is an important step towards the development of a cost-effective and high-yielding process for biodiesel production from lignocellulosic waste.

## Additional file

**Additional file 1: Table S1.** One-factor-at-a-time experimental design for optimization of lipid yield in newly isolated marine oleaginous yeasts.

## References

[CR1] Ageitos JM, Vallejo JA, Veiga-Crespo P, Villa TG (2011). Oily yeasts as oleaginous cell
factories. Appl Microbiol Biotechnol.

[CR2] Angerbauer C, Siebenhofer M, Mittelbach M, Guebitz GM (2008). Conversion of sewage sludge into lipids by *Lipomyces starkeyi* for biodiesel
production. Bioresour Technol.

[CR3] Areesirisuk A, Chiu CH, Yen TB, Liu CH, Guo JH (2015). A novel oleaginous yeast strain with high lipid
productivity and its application to alternative biodiesel
production. Appl Biochem Microbiol.

[CR4] Béligon V, Poughon L, Christophe G, Lebert A, Larroche C, Fontanille P (2015). Improvement and modeling of culture parameters to
enhance biomass and lipid production by the oleaginous yeast *Cryptococcus curvatus* grown on acetate. Bioresour Technol.

[CR5] Braunwald T, Schwemmlein L, Graeff-Hönninger S, French WT, Hernandez R, Holmes WE, Claupein W (2013). Effect of different C/N ratios on carotenoid and lipid
production by *Rhodotorula glutinis*. Appl Microbiol Biotechnol.

[CR6] Cescut J, Fillaudeau L, Molina-Jouve C, Uribelarrea JL (2014). Carbon accumulation in *Rhodotorula glutinis* induced by nitrogen limitation. Biotechnol Biofuels.

[CR7] Deeba F, Pruthi V, Negi YS (2016). Converting paper mill sludge into neutral lipids by
oleaginous yeast *Cryptococcus vishniaccii* for
biodiesel production. Bioresour Technol.

[CR8] Dias C, Sousa S, Caldeira J, Reis A, Lopes da Silva T (2015). New dual-stage pH control fed-batch cultivation
strategy for the improvement of lipids and carotenoids production by the red
yeast *Rhodosporidium toruloides* NCYC
921. Bioresour Technol.

[CR9] Gao Q, Cui Z, Zhang J, Bao J (2014). Lipid fermentation of corncob residues hydrolysate by
oleaginous yeast *Trichosporon cutaneum*. Bioresour Technol.

[CR10] Ghanavati H, Nahvi I, Roghanian R (2014). Monitoring growth and lipid production of new isolated
oleaginous yeast *Cryptococcus aerius* UIMC65
on glucose and xylose cultures. Biotechnol Bioprocess Eng.

[CR11] Ghanavati H, Nahvi I, Karimi K (2015). Organic fraction of municipal solid waste as a
suitable feedstock for the production of lipid by oleaginous yeast *Cryptococcus aerius*. Waste Manag.

[CR12] Granger L, Perlot P, Goma G, Pareilleux A (1993). Efficiency of fatty acid synthesis by oleaginous
yeasts: prediction of yield and fatty acid cell content from consumed C/N ratio
by a simple method. Biotechnol Bioeng.

[CR13] Johnson V, Singh M, Saini VS, Sista VR, Yadav NK (1992). Effect of pH on lipid accumulation by an oleaginous
yeast: *Rhodotorula glutinis*
IIP-30. World J Microbiol Biotechnol.

[CR14] Kaneko H, Hosohara M, Tanaka M, Itoh T (1976). Lipid composition of 30 species of
yeast. Lipids.

[CR15] Kimura K, Yamaoka M, Kamisaka Y (2004). Rapid estimation of lipids in oleaginous fungi and
yeasts using Nile red fluorescence. J Microbiol Methods.

[CR16] Kitcha S, Cheirsilp B (2013). Enhancing lipid production from crude glycerol by
newly isolated oleaginous yeasts: strain selection, process optimization, and
fed-batch strategy. Bioenergy Res.

[CR17] Kutty SN, Philip R (2008). Marine yeasts-a review. Yeast.

[CR18] Lepage G, Roy CC (1984). Improved recovery of fatty acid through direct
transesterification without prior extraction or purification. J Lipid Res.

[CR19] Li L, Singh P, Liu Y, Pan S, Wang G (2014). Diversity and biochemical features of culturable fungi
from the coastal waters of Southern China. AMB Express.

[CR20] Meesters PAEP, Van Der Wal H, Weusthuis R, Eggink G (1996). Cultivation of the oleaginous yeast *Cryptococcus Curvatus* in a new reactor with improved
mixing and mass transfer characteristics
(surer^®^). Biotechnol Tech.

[CR21] Papanikolaou S, Aggelis G (2011). Lipids of oleaginous yeasts. part I: biochemistry of
single cell oil production. Eur J Lipid Sci Technol.

[CR22] Polburee P, Yongmanitchai W, Lertwattanasakul N, Ohashi T, Fujiyama K, Limtong S (2015). Characterization of oleaginous yeasts accumulating
high levels of lipid when cultivated in glycerol and their potential for lipid
production from biodiesel-derived crude glycerol. Fungal Biol.

[CR23] Poli JS, da Silva MAN, Siqueira EP, Pasa VMD, Rosa CA, Valente P (2014). Microbial lipid produced by *Yarrowia lipolytica* QU21 using industrial waste: a potential
feedstock for biodiesel production. Bioresour Technol.

[CR24] Rakicka M, Lazar Z, Dulermo T, Fickers P, Nicaud JM (2015). Lipid production by the oleaginous yeast *Yarrowia lipolytica* using industrial by-products
under different culture conditions. Biotechnol Biofuels.

[CR25] Seo YH, Han S, Han JI (2014). Economic biodiesel production using algal residue as
substrate of lipid producing yeast *Cryptococcus
curvatus*. Renew Energy.

[CR26] Shen H, Gong Z, Yang X, Jin G, Bai F, Zhao ZK (2013). Kinetics of continuous cultivation of the oleaginous
yeast *Rhodosporidium toruloides*. J Biotechnol.

[CR27] Sitepu IR, Sestric R, Ignatia L, Levin D, German JB, Gillies LA, Almada LAG, Boundy-Mills KL (2013). Manipulation of culture conditions alters lipid
content and fatty acid profiles of a wide variety of known and new oleaginous
yeast species. Bioresour Technol.

[CR28] Sitepu IR, Garay LA, Sestric R, Levin D, Block DE, German JB, Boundy-Mills KL (2014). Oleaginous yeasts for biodiesel: current and future
trends in biology and production. Biotechnol Adv.

[CR29] Slininger PJ, Dien BS, Kurtzman CP, Moser BR, Bakota EL, Thompson SR, O’Bryan PJ, Cotta MA, Balan V, Jin M, Sousa LdC, Dale BE (2016). Comparative lipid production by oleaginous yeasts in
hydrolyzates of lignocellulosic biomass and process strategy for high
titers. Biotechnol Bioeng.

[CR30] Smit E, Leeflang P, Glandorf B, van Elsas JD, Wernars K (1999). Analysis of fungal diversity in the wheat rhizosphere
by sequencing of cloned PCR-amplified genes encoding 18S rRNA and temperature
gradient gel electrophoresis. Appl Environ Microbiol.

[CR31] Spier F, Buffon JG, Burkert CA (2015). Bioconversion of raw glycerol generated from the
synthesis of biodiesel by different oleaginous yeasts: lipid content and fatty
acid profile of biomass. Indian J Microbiol.

[CR32] Tamura K, Stecher G, Peterson D, Filipski A, Kumar S (2013). MEGA6: molecular evolutionary genetics analysis
version 6.0. Mol Biol Evol.

[CR33] Tanimura A, Takashima M, Sugita T, Endoh R, Kikukawa M, Yamaguchi S, Sakuradani E, Ogawa J, Ohkuma M, Shima J (2014). *Cryptococcus terricola* is a promising
oleaginous yeast for biodiesel production from starch through consolidated
bioprocessing. Sci Rep.

[CR34] Tanimura A, Takashima M, Sugita T, Endoh R, Kikukawa M, Yamaguchi S, Sakuradani E, Ogawa J, Shima J (2014). Selection of oleaginous yeasts with high lipid
productivity for practical biodiesel production. Bioresour Technol.

[CR35] Wang X, Ren H (2014). Microbial oil production by *Rhodotorula glutinis* CICC 31643 using sugar cane
molasses. J Renew Sustain Energy.

[CR36] Xiong L, Huang C, Yang XY, Lin XQ, Chen XF, Wang C, Wang B, Zeng XA, Chen XD (2015). Beneficial effect of corncob acid hydrolysate on the
lipid production by oleaginous yeast *Trichosporon
dermatis*. Prep Biochem Biotechnol.

[CR37] Yang X, Jin G, Gong Z, Shen H, Bai F, Zhao ZK (2014). Recycling biodiesel-derived glycerol by the oleaginous
yeast *Rhodosporidium toruloides* Y4 through
the two-stage lipid production process. Biochem Eng J.

[CR38] Zaky AS, Tucker GA, Daw ZY, Du C (2014). Marine yeast isolation and industrial
application. FEMS Yeast Res.

[CR39] Zhang X, Shen H, Yang X, Wang Q, Yu X, Zhao ZK (2016). Microbial lipid production by oleaginous yeasts on
Laminaria residue hydrolysates. RSC Adv.

