# Supplementary material for: Characterization and robust nature of newly isolated oleaginous marine yeast Rhodosporidium spp. from coastal water of Northern China
Source: AMB Express. 2017 Feb 1;7:30. doi: 10.1186/s13568-017-0329-x (PMC5302000; doi:10.1186/s13568-017-0329-x)
Supplement: Supplementary file 1 — Additional file 1: Table S1. One-factor-at-a-time experimental design for optimization of lipid yield in newly isolated marine oleaginous yeasts. [file 13568_2017_329_MOESM1_ESM.docx]

Supplementary Information for

**Characterization and Robust Nature of Newly Isolated Oleaginous Marine Yeast *Rhodosporidium* *spp*. from Coastal Water of Northern China**

Qiuzhen Wang^a§^, Yan Cui^b§^, Biswarup Sen^a^, Wenmeng Ma^a^, Rose Lynn Zheng^a^, Xianhua Liu^a^, Guangyi Wang^a^*

*To whom correspondence should be addressed.

Email: [gywang@tju.edu.cn](mailto:gywang@tju.edu.cn)

**Supplemental Table 1**: One-factor-at-a-time experimental design for optimization of lipid yield in newly isolated marine oleaginous yeasts

| **Design steps** | **Factors** | | | | |
| --- | --- | --- | --- | --- | --- |
|  | **Type of carbon source** | **Nitrogen source** | **pH** | **T (**°C**)** | **Salinity**  **(% v/v)** |
| Step 1: Carbon source | 1. Glucose 2. Sucrose 3. Fructose 4. Lactose 5. Starch   Conc.: 120 g/L | NH_4_Cl (0.5 g/l)  + YE (1.5 g/l) | 6 | 28 °C | 100% |
| Step 2: Nitrogen source | Glucose  (120 g/L) | 1. *Single*   1. Peptone  2. Yeast Extract (YE)  3. NH_4_Cl  4. (NH_4_)_2_SO_4_  Conc.: 2g/L   1. *Combination*   5. (NH_4_)_2_SO_4_ (0.5 g/l) + YE (1.5 g/l)  6. (NH_4_)_2_SO_4_ (1.5 g/l) + Peptone (0.5 g/l)  7. NH_4_Cl (0.5 g/l) + YE (1.5 g/l)  8. NH_4_Cl 1.5 g/l + Peptone (0.5 g/l)  9. YE (1.5 g/l) + Peptone (0.5 g/l) | 6 | 28 °C | 100% |
| Step 3:  pH | Glucose  (120 g/L) | YE (1.5 g/l) + Peptone (0.5 g/l) | 3.0, 4.0, 5.0, 6.0, and 7.0 | 28 °C | 100% |
| Step 4: Temperature | Glucose  (120 g/L) | YE (1.5 g/l) + Peptone (0.5 g/l) | 6 | 15, 20, 25, 30 and 35 °C | 100% |
| Step 5: Salinity | Glucose  (120 g/L) | YE (1.5 g/l) + Peptone (0.5 g/l) | 6 | 15, 20, 25, 30 and 35 °C | 30, 50, 80 and 100 % |
